# Effects of Environmental Temperature on the Dynamics of Ichthyophoniasis in Juvenile Pacific Herring (*Clupea pallasii*)

**DOI:** 10.1155/2011/563412

**Published:** 2011-04-26

**Authors:** Jake L. Gregg, Johanna J. Vollenweider, Courtney A. Grady, Ron A. Heintz, Paul K. Hershberger

**Affiliations:** ^1^U.S. Geological Survey, Western Fisheries Research Center, Marrowstone Marine Field Station, 616 Marrowstone Point Road, Nordland, WA 98358, USA; ^2^NOAA National Marine Fisheries Service, Alaska Fisheries Science Center, Auke Bay Laboratories, 17109 Pt. Lena Loop Road, Juneau, AK 99801, USA

## Abstract

The effects of temperature and infection by *Ichthyophonus* were examined in juvenile Pacific herring (*Clupea pallasii*) maintained under simulated overwinter fasting conditions. In addition to defining parameters for a herring bioenergetics model (discussed in Vollenweider et al. this issue), these experiments provided new insights into factors influencing the infectivity and virulence of the parasite *Ichthyophonus*. In groups of fish with established disease, temperature variation had little effect on disease outcome. *Ichthyophonus* mortality outpaced that resulting from starvation alone. In newly infected fish, temperature variation significantly changed the mortality patterns related to disease. Both elevated and lowered temperatures suppressed disease-related mortality relative to ambient treatments. When parasite exposure dose decreased, an inverse relationship between infection prevalence and temperature was detected. These findings suggest interplay between temperature optima for parasite growth and host immune function and have implications for our understanding of how *Ichthyophonus* infections are established in wild fish populations.

## 1. Introduction

*Ichthyophonus hoferi*, a highly pathogenic parasite of marine and anadromous fishes, is currently ubiquitous in Pacific herring (*Clupea pallasii*) populations throughout the NE Pacific [1–3]. Phenotypic [4] and genotypic [5, 6] differences among isolates of *I. hoferi* suggest that there are multiple sympatric species in the region. Due to this taxonomic uncertainty, here we refer to the parasite by its generic name. Among wild Pacific herring, the prevalence of infection typically increases with age; consequently, the resulting disease (ichthyophoniasis) is thought to affect primarily older age cohorts [2, 7]. However, the impact of *Ichthyophonus* infections on juvenile Pacific herring has received little attention. In Atlantic herring (*Clupea harengus*), it has been suggested that time to mortality is shorter for juveniles than for adults resulting in relatively low, but highly variable, prevalence [8]. Prevalence of infection among young-of-the-year (YOY) herring is typically 3–13% in the NE Pacific (USGS unpublished data) with infections detected as early as 4 months after hatch [9]. In addition to causing direct mortality from disease, *Ichthyophonus* infection may predispose juvenile herring to indirect mortality through predation [10], presumably as a result of decreased swimming performance [11, 12]. This type of indirect mortality is difficult to demonstrate but should be expected considering the extensive tissue damage that can result from ichthyophoniasis. *Ichthyophonus* invades multiple visceral organs and can appear in the heart within 26 h after exposure [13]. Kocan et al. [11] reported a 40% increase in cardiac weight in infected rainbow trout (*Oncorhynchus mykiss*) resulting from parasite biomass and host inflammatory response. The physiological cost of ichthyophoniasis and resulting reduction in fitness could compound the effects of other environmental and biological stressors experienced by juvenile Pacific herring.

In the NE Pacific, specifically Prince William Sound, the overwinter fast has been identified as a primary factor limiting YOY herring survival [14]. This study and its companion (see in Vollenweider et al. this issue) were designed to improve our understanding of the bioenergetic costs of overwinter fasting for juvenile Pacific herring. We were specifically interested in the compounding effects of disease, temperature, and starvation on survival, and the effect of temperature on disease processes. Three experiments were conducted to determine (1) effects of temperature on mortality in fasting Pacific herring with established disease, (2) effects of temperature on disease progression and mortality in fasting Pacific herring immediately after parasite exposure, and (3) effects of temperature on disease progression and mortality in Pacific herring recovering from pre-exposure fast.

## 2. Materials and Methods

### 2.1. Pacific Herring

To ensure that animals were parasite-free and immunologically naïve at initiation of experiments, a cohort of specific-pathogen-free (SPF) Pacific herring was reared for this study at the USGS Marrowstone Marine Field Station (MMFS) (Nordland, WA, USA). Naturally spawned eggs from wild Pacific herring were collected from the southern Strait of Georgia (48°55.85′N, 122°48.15′W) on 5 May 2008 and transported to MMFS where they were held in 270 L tanks supplied with filtered, UV-irradiated seawater. Eggs hatched on 18 May 2008 and larvae were subsequently moved to 1700 L tanks where they were fed enriched live feeds (i.e., *Brachionus plicatilis* and *Artemia franciscana*) and frozen copepods (Cyclop-eeze, Argent Chemical Laboratories, Redmond, WA, USA) until metamorphosis to juveniles. Juvenile herring were weaned to a krill-meal pellet produced at the USFWS Abernathy Fish Technology Center (Longview, WA, USA) and eventually to commercially available salmon pellet (Bio-Olympic Fry, Bio-Oregon, Longview, WA, USA). 

### 2.2. Experiment Design and Environment

Three experiments were performed over an eleven-month period to investigate the effects of fasting and temperature on disease dynamics in *Ichthyophonus*-infected Pacific herring (Table 1). Two treatments, infected and uninfected, were applied to fasting herring held at each of three temperatures (low, ambient, and high). To control for the effect of fasting, this 2 × 3 design was modified with the addition of two fed treatments, infected and uninfected, maintained at ambient temperature. Herring were randomly assigned to triplicate 270 L tanks in each treatment. Flow through seawater (low, ambient, and high temperature) was delivered to each tank at the rate of 4 L min^−1^. The temperature regulating system allowed temperatures to vary with seasonal seawater temperature, but maintained a relatively consistent separation between low, ambient, and high temperature treatments. Seawater temperatures in low and high treatments were adjusted gradually over the first 24 h after fish were distributed to replicate tanks. Water temperature was recorded every 30 min (Hobo Water Temp Pro v2, Onset Computer Corp., Bourne, MA, USA). Temperature treatments will be identified here as the mean temperature that occurred throughout the experimental period.

### 2.3. Parasite Exposure

*Ichthyophonus* exposure for herring in infected treatments occurred via intraperitoneal (IP) injection. To obtain stock material for *Ichthyophonus* inoculum, heart tissues from *Ichthyophonus*-infected herring (wild and laboratory infected) were cultured in Eagle's minimum essential medium supplemented with fetal bovine serum (5% v/v), penicillin (100 IU mL^−1^), streptomycin (100 *μ*g mL^−1^) and gentamycin (100 *μ*g mL^−1^) and buffered to pH 7.8 with 1 M Tris (MEM). After 2–4 weeks in culture, thousands of *Ichthyophonus *schizonts (i.e., spherical multinucleate bodies) had grown out of the tissues and were free in the media. Host tissues and medium were removed from these cultures with sterile pipettes, several cultures were combined, and this mixture was diluted in sterile phosphate-buffered saline (PBS) to produce an inoculating solution that contained approximately 2000 schizonts mL^−1^. Fifty *μ*L of inoculum was then injected into each fish with the goal of introducing 100 schizonts/fish regardless of size. For each experiment, three 50 microliter aliquots were collected during the injection process and schizonts were counted at 40X magnification on an inverted microscope. Final inoculum concentration (i.e. dose) for each experiment was determined by dividing the mean number of schizonts by mean fish weight.

### 2.4. Effects of Temperature on Mortality in Fasting Pacific Herring with Established Disease

Juvenile SPF herring (age = 145 d) were inoculated with *Ichthyophonus* schizonts (mean = 174 schizonts fish^−1^) and placed in a 760 L holding tank. Nineteen days post-exposure (DPE), when external signs of ichthyophoniasis (i.e. black skin ulcerations) were evident in many individuals, herring from this tank and a corresponding tank containing uninfected controls were distributed to triplicate 260 L tanks (*n* = 47 to 52 fish/tank) at each of three temperatures, 6.7°C (low), 9.3°C (ambient), and 12.3°C (high), and fasting was initiated. Additional 9.3°C (ambient) treatment groups with infected and uninfected herring were fed to satiation daily. The experiment was terminated 80 DPE.

### 2.5. Effects of Temperature on Disease Progression and Mortality in Fasting Pacific Herring Immediately after Parasite Exposure

Juvenile SPF herring (age = 241 d) were injected with *Ichthyophonus* schizonts (mean = 167 schizonts fish^−1^) or PBS and immediately distributed to triplicate tanks (*n* = 49 to 51 fish/tank) at each of three temperatures: 5.6°C (low), 7.9°C (ambient), and 12.4°C (high), and fasting was initiated. Additional 7.9°C (ambient) treatment groups with infected and uninfected herring were fed to satiation daily. Treatments were terminated between 111 and 127 DPE.

### 2.6. Effects of Temperature on Disease Progression and Mortality in Pacific Herring Recovering from Pre-Exposure Fast

Feed was withheld from 607 SPF herring held in two 760 L tanks and a second group of 300 SPF herring in a single 760 L tank were fed to excess 3x week^−1^ (i.e., >10% biomass per feeding). The experiment was initiated after 56 d when herring from the fasted colony were injected with either *Ichthyophonus* or PBS and transferred to triplicate tanks (*N* = 27 to 30 fish per tank) at each of three temperatures: 9.3°C (low), 12.0°C (ambient), and 15.3°C (high). Herring from the fed colony were injected with either *Ichthyophonus *or PBS and transferred to triplicate tanks at 12.0°C (ambient). Fish in all treatments (previously fasted and fed) were fed to satiation daily after exposure. Live herring were subsampled (*n* = 2 tank^−1^) for bioenergetic analysis (see in Vollenweider et al. this issue) 31 DPE, and the experiment was terminated 54 DPE. 

### 2.7. Fish Sampling

A subsample (*n* = 20 fish) was taken from pre-experiment pools for fork length (FL) and weight (WT) measurements at the initiation of each experiment. Mortalities in all challenges were removed from tanks daily, and survivors were euthanized with an overdose of tricaine methane sulfonate in buffered seawater at the termination of each experiment. Prevalence of infection and disease was determined among mortalities and survivors from all experiments. Prevalence of infection was determined by *in vitro* explant culture of heart tissue in MEM. Heart cultures were incubated at 15°C and examined microscopically (40x magnification) for the presence of *Ichthyophonus* schizonts and/or hyphae after 14 d. Culture-positive samples were considered diseased when visible lesions indicative of ichthyophoniasis were observed on the skin and/or heart.

### 2.8. Statistics

Cumulative mortality was compared between treatments at the midpoint and end of each experiment using a single factor (treatment) ANOVA followed by a Tukey Test for multiple comparisons. Infection and disease prevalence were similarly compared between the *Ichthyophonus*-exposed treatments. All analyses were conducted using arcsine transformed data. Statistical significance was assigned to comparisons with *P* ≤ .05.

## 3. Results

### 3.1. Mortality in Fasting Pacific Herring with Established Disease

Herring were 44 mm FL (SD = 4.7) and 1.0 g WT (SD = 0.38) at the initiation of this challenge. A dose of 174 schizonts g^−1^ successfully established *Ichthyophonus* infections, and a majority of the infections progressed to overt disease. In *Ichthyophonus*-exposed groups, infection prevalence ranged from 95.9 to 98.6% with no significant differences between treatments (*F*_0.05(2),3,8_ = 1.749, *P* < .23). Similarly, prevalence of disease (i.e., clinical signs) among infected fish ranged from 97.0 to 99.3% with no significant differences between treatments (*F*_0.05(2),3,8_ = 1.089, *P* < .41). *Ichthyophonus* was not detected in any herring from unexposed control groups.

**Figure 1:**
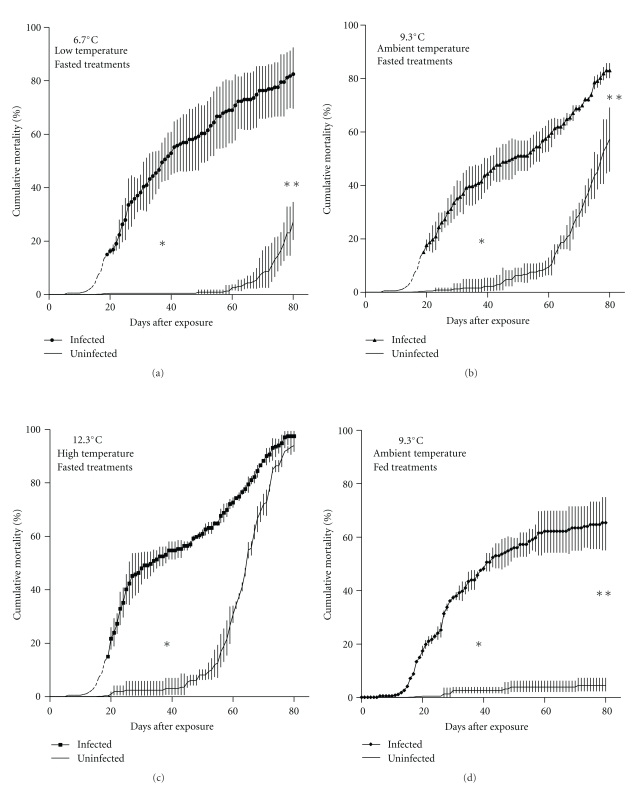
*Clupea pallasii*. Cumulative mortality of diseased (ichthyophoniasis) and control Pacific herring in three temperature treatments. Data in (a), (b), and (c) are from fish undergoing simulated overwinter fast. Data in (d) are from fed treatments. Temperature adjustments were made 19 days after inoculation with *Ichthyophonus* schizonts. Data are means of 3 replicate tanks in each treatment. Error bars are one SD above and below the mean. Means and SD were calculated from arcsine-transformed data. Mortality data prior to day 19 are from the pools of infected and control fish prior to separation into treatment. Significant differences between infected and uninfected groups (Tukey multiple comparisons) indicated by ∗ and ∗∗ for midpoint and end of challenge, respectively.

Cumulative mortality in infected treatments generally followed a sigmoid pattern reaching 44% to 55% by the midpoint of the experiment and 65% to 98% by the end (Figure 1). Cumulative mortality varied significantly with treatment at the midpoint of the experiment (day 40; *F*_0.05(2),7,16_ = 102.2, *P* < 4.4 × 10^−12^). The Tukey test for multiple comparisons grouped all infected treatments separate from uninfected controls on 40 DPE, with no separation due to temperature. Starvation mortality in fasting, uninfected treatments followed a predictable pattern in relation to temperature. At 52, 61, and 72 DPE, cumulative mortality reached 10% in 12.3°C (high), 9.3°C (ambient), and 6.7°C (low) treatments, respectively, and increased exponentially until termination of the experiment (Figures 1(a), 1(b), and 1(c)). Starvation mortality continued in infected treatments late into the challenge preventing a plateau that appeared to be developing in infected treatments around 60% to 70%. This plateau did develop in the single infected treatment (9.3°C) that was fed throughout the challenge (Figure 1(d)). At termination of the experiment, significant differences in cumulative mortality did exist between treatments (day 80; *F*_0.05(2),7,16_ = 55.3, *P* < 5.0 × 10^−10^), but the Tukey test did not group the treatments by infection status as on day 40. At termination cumulative mortality in fasting uninfected treatments approached or surpassed that resulting from disease alone. Cumulative mortality in the uninfected, fed treatment (9.3°C) remained below 5% (Figure 1(d)).

### 3.2. Disease Progression and Mortality in Fasting Pacific Herring Immediately after Parasite Exposure

Herring were 70 mm FL (SD = 6.3) and 3.6 g WT (SD = 0.97) at the initiation of this challenge. A dose of 46 schizonts g^−1^ successfully established *Ichthyophonus* infections, and a majority of the infections progressed to overt disease. In *Ichthyophonus*-exposed groups, infection prevalence ranged from 95.4% to 98.0% with no significant differences between treatments (*F*_0.05(2),3,8_ = 1.005, *P* < .44). Similarly, prevalence of disease (i.e., clinical signs) among infected fish ranged from 95.1% to 99.3% with no significant differences between treatments (*F*_0.05(2),3,8_ = 1.977, *P* < .20). *Ichthyophonus* was not detected in any herring from PBS-injected control groups.

**Figure 2:**
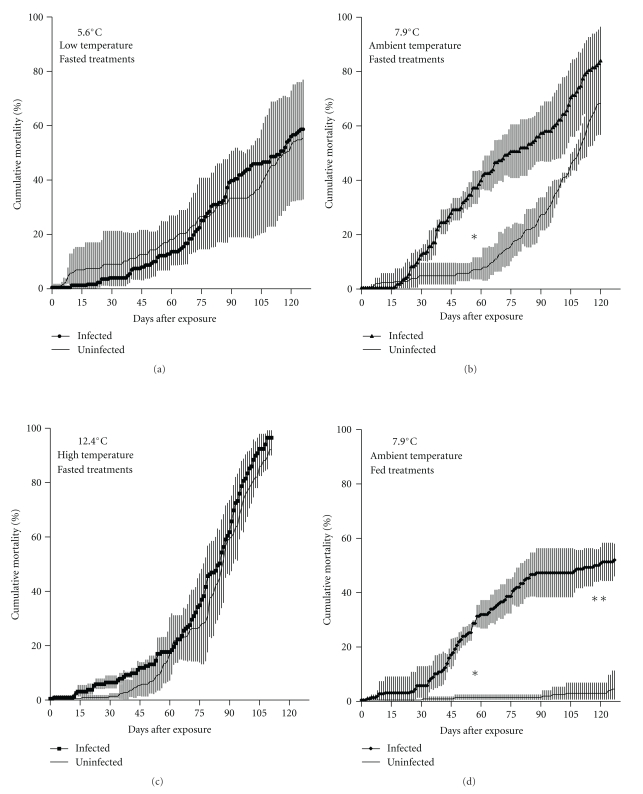
*Clupea pallasii*. Cumulative mortality of infected (*Ichthyophonus*) and control Pacific herring in three temperature treatments. Data in (a), (b), and (c) are from fish undergoing simulated overwinter fast. Data in (d) are from fed treatments. *Ichthyophonus* inoculation occurred on day 0, temperature adjustments on day 1. Data are means of 3 replicate tanks in each treatment. Error bars are one SD above and below the mean. Means and SD were calculated from arcsine-transformed data. Significant differences between infected and uninfected groups (Tukey multiple comparisons) indicated by ∗ and ∗∗ for midpoint and end of challenge, respectively.

Disease-related mortality was negated in fasting Pacific herring moved to 5.6°C and 12.4°C shortly after *Ichthyophonus* exposure (Figures 2(a) and 2(c)), while the newly exposed Pacific herring remaining at 7.9°C displayed mortality kinetics (Figures 2(b) and 2(d)) similar to fish with established disease (Figures 1(b) and 1(d)). Significant difference did occur between treatments at midpoint (*F*_0.05(2),7,16_ = 11.83, *P* < 2.8 × 10^−5^) and end (*F*_0.05(2),7,16_ = 30.45, *P* < 4.3 × 10^−8^) of the challenge, but these differences were not simply the result of differences between infected and uninfected treatments. Cumulative mortality at 5.6°C reached 48.7% and 45.3% in infected and uninfected treatments, respectively. Little separation occurred between these treatments at the midpoint or end of the experiment. Similarly, there were no significant differences between uninfected and infected treatments at 12.4°C, where mortality reached 92.0% and 96.5%, respectively, by the end of the experiment. Mortality curves in infected groups held at 5.6°C and 12.4°C were similar to those which resulted from starvation alone (Figures 2(a) and 2(c)), a simple exponential increase. Mortality in herring held at 7.9°C after exposure varied significantly with infection status (Figures 2(b) and 2(d)). Cumulative mortality in fed infected herring reached 25.3% 55 DPE and plateaued just below 50% around 90 DPE. Mortality of fasted infected herring reached 34.6% 55 DPE but did not plateau as starvation mortality (evident in uninfected groups) ensued, pushing cumulative mortality to 83.1% by the end of the experiment. Fed uninfected fish experienced less than 5% mortality. 

### 3.3. Disease Progression and Mortality in Pacific Herring Recovering from Pre-Exposure Fast

**Figure 3:**
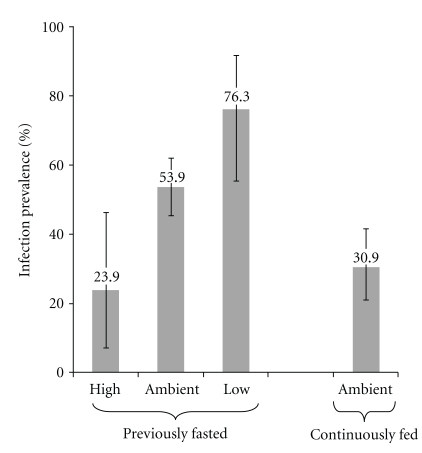
*Clupea pallasii. Ichthyophonus* infection prevalence in groups of Pacific herring held at three temperatures. Data are means of arcsine transformed data from 3 replicate tanks; error bars are one SD above and below the mean. Mean temperatures were 9.3°C, 12.0°C, and 15.3°C for low, ambient, and high treatments, respectively. Prechallenge fast lasted 56 days, after which fish were inoculated with *Ichthyophonus*, separated to treatments, and fed to satiation daily. ANOVA and Tukey test for multiple comparisons indicates that high-temperature treatment and continuously fed treatments are significantly different from low-temperature treatment.

Prevalence of *Ichthyophonus* infection after IP exposure to schizonts was inversely related to water temperature. Pre-exposure fasting resulted in herring that were 92 mm FL (SD = 11.7) and 6.5 g WT (SD = 2.4). A dose of 17 schizonts g^−1^ established infection in 76.3%, 53.9%, and 23.9% of these fish in 9.3°C, 12.0°C, and 15.3°C (low, ambient, and high) treatments, respectively. The pre-exposure fed group was larger (100 mm, SD = 12.3; 9.2 g, SD = 3.2) resulting in a lower dose of 12 schizonts g^−1^. This dose established infections in 30.9% of the fish at 12.0°C. Prevalence of infection varied significantly with treatment (Figure 3; *F*_0.05(2),3,8_ = 5.956, *P* < .02). Among infected herring, prevalence of disease (i.e., clinical signs) ranged from 95.0% to 100% with no significant differences between treatments (*F*_0.05(2),3,8_ = 0.668, *P* < .59). *Ichthyophonus* was not detected in any control herring injected with PBS. 

Mortality resulting from handling and injection of fish at the initiation of this experiment was very high, most likely due to the weakened condition of the fish at the end of the prechallenge fast, some treatments lost as many as 28% of fish in first few days after exposure. For this reason, we calculated cumulative mortality based on the fish that remained in the tanks on day 10 and only compared cumulative mortality (ANOVA) at the end of the challenge. Overall, adjusted cumulative mortality was lower and more variable than in the previous two challenges; 6.1% to 15.9% in uninfected treatments and 11.8% to 24.9% in infected treatments; ANOVA detected no significant differences between treatments (*F*_0.05(2),7,16_ = 2.23, *P* < .09).

## 4. Discussion

Disease processes do vary with temperature in juvenile Pacific herring infected with *Ichthyophonus*. However, the degree to which changes in temperature alter the outcome (i.e, infection prevalence or cumulative mortality) for *Ichthyophonus*-infected groups depends on the stage/intensity of the infection. Temperature manipulation had no detectable effect on *Ichthyophonus* infections that had progressed to overt disease. Mortality in these cases outpaced that which resulted from starvation alone (Figure 1). However, disease outcome was affected when temperature manipulation occurred within 24 h of *Ichthyophonus* exposure. Cumulative mortality in high (12.4°C) and low (5.6°C) temperature infected treatments was not significantly different from that of corresponding uninfected groups, while mortality kinetics in ambient treatments (7.9°C) did differ with infection status (Figure 2). Suppression of *Ichthyophonus* mortality was so complete in high and low temperatures that net *Ichthyophonus* mortality (i.e., disease mortality—control mortality) remained low throughout the study and at some time points was negative. Slower parasite growth could explain the lower mortality in low temperature treatments, as our experiments were conducted in a range where temperature and *Ichthyophonus* growth (*in vitro*) are directly related [15, 16]. However, if the relationship was simply based on parasite growth, the trend would hold and mortality would increase with increasing temperature, as has been demonstrated in some bacteria-salmonid systems [17, 18]. Suppressed mortality from ichthyophoniasis in both low and high temperature treatments suggests interplay of temperature optima for parasite growth and host immune function. 

Innate and adaptive immune functions of fish are significantly affected by environmental temperature [19–22]. Immunocompetence is generally considered to vary directly with temperature within the homeostatic range of the host; however, there is suggestion that at low temperatures some nonspecific immune functions may increase to compensate for suppression of adaptive immune responses [22]. Complement-mediated lytic activity [23], production of macrophage activating factor [24], natural antibody activity [19], and protection resulting from vaccination [25] have all been shown to increase with increased temperature. The immune response to histozoic parasites includes an innate response at the mucous layer, an innate response in tissues, and eventually an adaptive response in tissues [26, 27]. *Ichthyophonus* has been shown to elicit a cell mediate innate immune response in plaice (*Pleuronectes platessa*), haddock (*Melanogrammus aeglefinus*), and rainbow trout (*O. mykiss*) with persistent infections resulting in focal granulomata that encapsulate the parasite [13]. An antibody response has been demonstrated in plaice, but there is no evidence that it is protective [28].

The suppression of *Ichthyophonus* by a temperature-enhanced innate response is a plausible explanation for the reduced *Ichthyophonus* mortality that we observed in the newly exposed high-temperature treatment. This hypothesis is further supported by prevalence data from the pre-exposure fast challenge, where 76%, 54%, and 24% of fish were infected in the low, ambient, and high temperature fasted/fed, treatments, respectively (Figure 3). The relative dose of *Ichthyophonus* decreased over the course of this study as fish mass increased (Table 1). In the third challenge, fasted fish received 17 schizonts g^−1^. At this dose, a fraction of the fish were able to clear the schizonts and prevent establishment of infection, and the number that was able to do so was markedly (3x) higher in the high-temperature treatment. This phenomenon was not evident in the second challenge where inoculation dose was 46 schizonts g^−1^, suggesting that high-parasite load can mask temperature effects. The importance of parasite load/dose in determining disease outcome is also evident in comparison of ambient treatments in the third challenge. Previously fasted fish received 17 schizonts g^−1^, while continuously fed received 12 schizonts g^−1^, resulting in mean infection prevalence of 54% and 31%, respectively (Figure 3). 

The temperature-driven variation demonstrated in this study has implications for our understanding of disease processes in wild herring populations. The temperatures used (5.6°C to 15.3°C) are within those that the host species can experience across its geographic distribution [29–32], and the magnitude of temperature variation used in each challenge (5-6°C) could plausibly be experienced by a single individual as the result of season temperature fluctuation and migration [14, 33]. We did not see temperature effects in diseased fish but did see differences at early stages of infection, especially when exposure dose was low, suggesting that seawater temperature at time of exposure could be an important factor that determines disease prevalence over ensuing months and years. Unfortunately, the complete life cycle of *Ichthyophonus* is not known, and whether or not the disease is monoxenous in Pacific herring is still unclear. Other species such as rainbow trout, haddock, and plaice can become infected by eating infected fish tissues [11, 13], but the route of infection in Pacific herring is still in question. Juveniles captured shortly after metamorphosis are infected when they are still obligate planktivores [9]. Future field surveys and empirical studies should focus on closing the life history gaps that exist for *Ichthyophonus*, specifically the modality and timing of infection in Pacific herring. Once these variables are understood, more focused work could incorporate the findings of this study to mimic possible environmental processes at the time of infection.

## Figures and Tables

**Table 1:** *Clupea pallasii*. Summary data for 3 *Ichthyophonus* experiments conducted in this study. Pacific herring in experiments 1 and 2 fasted during challenge, herring in experiment 3 fasted for 56 d prior to challenge and then fed to satiation during challenge.

	Experiment		Herring			*Ichthyophonus*		Temperature (°C)^a^		
No.	Disease state	Duration (d)	Age (d)	FL (mm)	Weight (g)	Schizonts^b^ per inoculation	Dose (schizonts g^−1^)^c^	Low	Amb	High
(1)	Diseased: 19 d after exposure	80	164	44	1.0	174	174	6.7	9.3	12.3
(2)	Infected: 1 d after exposure	127	241	70	3.6	167	46	5.6	7.9	12.4
(3)	Infected: 1 d after exposure	54	431	92 (100)^d^	6.5 (9.2)^d^	112	17 (12)^d^	9.3	12.0	15.3

^a^Temperature reported is mean of temperature recordings made every 30 minutes during course of challenge.

^b^Schizonts are multinucleate spherical bodies from *in vitro Ichthyophonus *cultures, number reported is mean of three 50 *μ*L samples.

^c^Dose = mean number of schizonts divided by mean weight of fish.

^d^Values in parentheses are from group of herring that did not go through prechallenge fast in experiment (3).
